# Comparative Investigation of Water-Based CMC and LA133 Binders for CuO Anodes in High-Performance Lithium-Ion Batteries

**DOI:** 10.3390/molecules29174114

**Published:** 2024-08-30

**Authors:** Nischal Oli, Sunny Choudhary, Brad R. Weiner, Gerardo Morell, Ram S. Katiyar

**Affiliations:** 1 Department of Physics, University of Puerto Rico-Rio Piedras Campus, San Juan, PR 00925, USA; 2 Department of Chemistry, University of Puerto Rico-Rio Piedras Campus, San Juan, PR 00925, USA

**Keywords:** CuO, sodium carboxymethyl cellulose, carbon matrix, lithium-ion batteries, electrochemical reactions

## Abstract

Transition metal oxides are considered to be highly promising anode materials for high-energy lithium-ion batteries. While carbon matrices have demonstrated effectiveness in enhancing the electrical conductivity and accommodating the volume expansion of transition metal oxide-based anode materials in lithium-ion batteries (LIBs), achieving an optimized utilization ratio remains a challenging obstacle. In this investigation, we have devised a straightforward synthesis approach to fabricate CuO nano powder integrated with carbon matrix. We found that with the use of a sodium carboxymethyl cellulose (CMC) based binder and fluoroethylene carbonate additives, this anode exhibits enhanced performance compared to acrylonitrile multi-copolymer binder (LA133) based electrodes. CuO@CMC electrodes reveal a notable capacity ~1100 mA h g^−1^ at 100 mA g^−1^ following 170 cycles, and exhibit prolonged cycling stability, with a capacity of 450 mA h g^−1^ at current density 300 mA g^−1^ over 500 cycles. Furthermore, they demonstrated outstanding rate performance and reduced charge transfer resistance. This study offers a viable approach for fabricating electrode materials for next-generation, high energy storage devices.

## 1. Introduction

In the incessant pursuit of greener and more highly efficient energy solutions, lithium-ion batteries (LIBs) have become an indispensable part of modern life and revolutionized energy storage technologies, powering portable electronics to electric vehicles (EVs) or hybrid electric vehicles (HEVs), due to their reasonable energy density (150–200 W h kg^−1^) and exceptional cycling stability [1,2,3,4,5]. Given the escalating demands for long-range electric vehicles, it is imperative to engineer batteries with superhigh energy densities (350–500 Wh kg^−1^) [6,7,8,9,10]. Lithium metal is one of the best selections for the anode material in next-generation lithium-air (3862 mA h g^−1^) [11], lithium-sulfur (1675 mA h g^−1^) [12], lithium metal batteries and solid-state lithium-based batteries, which have drawn worldwide attention within the battery community, owing to their high theoretical specific capacity of 3860 mA h g^−1^ and low potential of −3.04 V vs. the standard hydrogen electrode (SHE) [13]. Nevertheless, there are critical questions that need to be addressed before lithium metal can be commercially adopted as an anode on a widespread scale. For example, lithium metal is susceptible to dendrite formation, resulting in cell short-circuits and low Coulombic efficiency [14]. The persistent solid–solid interface issue in solid-state electrolytes poses a substantial challenge to contemporary battery manufacturing technology. Thus, the continued development of LIBs with enhanced energy densities remains an appealing option, until the full commercialization of lithium metal or solid-state batteries occurs.

The energy density of a battery primarily depends on the specific capacity of its electrode materials. Presently, existing commercial graphite stands as the dominant anode material for lithium-ion batteries (LIBs), owing to its low operational potential (~0.1 V vs. Li/Li^+^), cost-effectiveness, and robust cycling stability [15,16]. However, graphite faces several drawbacks. Mainly, its modest specific capacity of 372 mA h g^−1^ limits the energy density of LIBs. Another drawback is that graphite’s Li-insertion potential (~0.1 V vs. Li/Li^+^) approximates that of Li metal plating (~0 V vs. Li/Li^+^), which raises safety concerns, especially during fast charging and in low-temperature environments [15,17]. Nevertheless, these promising battery systems encounter inherent difficulties that are hard to overcome quickly. Hence, there is pressing demand to innovate high-performance anode materials, possessing a higher capacity, appropriate reaction potential, and environmentally sustainable materials [18,19].

Over the past two decades, there have been tremendous efforts using alloy-based materials in the search for suitable anode materials, notably silicon (Si) (4200 mA h g^−1^) [20], tin (Sn) (993 mA h g^−1^) [21], aluminum (Al) (993 mA h g^−1^) [22], antimony (Sb) (600 mA h g^−1^) [23], Germanium (Ge) (1624 mA h g^−1^) [24], and Bismuth (Bi) (385 mA h g^−1^). [25] Among these, silicon-based anode materials have gained global attention from research communities and manufacturers, owing to their potential for nearly tenfold greater specific (gravimetric) capacity compared to commercial graphite anodes (Si~4200 mA h g^−1^ vs. 372 mA h g^−1^ for graphite), but these silicon-based anode materials undergo a volumetric expansion of up to 300% during lithiation and delithiation processes [26,27]. However, mitigating the substantial capacity degradation of silicon electrodes poses a complex and multifaceted challenge. This capacity fading primarily stems from two distinct factors: primarily, silicon grain disintegration and fracture of the electrode integration, leading to a broken electrical contact with the active material’s current collector, and an unstable solid–electrolyte interphase (SEI) that results in electrolyte deformation. Similarly, all other alloy materials often experience significant volume expansion (100–300%), leading to the formation of SEI cracks, thereby compromising cycling stability [28]. There have been extensive efforts made to tackle these issues. Furthermore, there are also several anode materials that undergo a conversion-alloy reaction, involving an initial conversion followed by an alloy reaction. Oxides with high theoretical capacities, namely tin oxide (SnO_2_) (1490 mA h g^−1^), silicon oxide (SiO_x_), and bismuth ferrite (BiFeO_3_) (770 mA h g^−1^) are common materials undergoing a conversion-alloy reaction [29,30,31]. Research indicates that these anode materials demonstrate improved cycling performance compared to pure alloy materials, owing to the formation of lithium oxide (Li_2_O), which effectively alleviates electrode volume changes [32].

On the other hand, conversion-based transition-metal (TM) oxide materials emerge as promising candidates for anode materials in lithium-ion batteries (LIBs). It is denoted as TM_x_O_y_ (where TM = Ti, V, Cr, Mn, Fe, Co, Ni, Cu, Zn, Mo, etc.). These oxides present high reversible capacities by undergoing a redox mechanism with lithium (TM_x_O_y_ + 2yLi = yLi_2_O + xTM) [33,34,35]. These materials exhibit 2–3 time greater specific capacity than commercial graphite anode. This phenomenon serves as motivation to optimize compounds within the conversion-based material family, aiming to foster the development of high-energy, high-rate capability, and sustainable materials for LIBs [34,36].

Copper oxide (CuO) is an interesting material, with a monoclinic structure and semiconducting properties, but with poor electrical conductivity, which impedes efficient charge transfer [37]. Additionally, CuO-based electrodes suffer from substantial volume expansion and dispersion of copper elements within the Li_2_O matrix during charge–discharge process, leading to significant mechanical stress and rapid deterioration in capacity [38]. Enhanced electrochemical performance of CuO-based anodes is crucial to meet the demands of next generation lithium-ion batteries (LIBs). Considerable efforts have been made to bolster the performance of CuO-based electrodes via morphology modification, designing nanostructured configuration and hybridization with composite materials, resulting in materials that exhibit promising electrochemical performance and distinctive structures [38,39,40,41]. This material distinguishes itself for its cost-effectiveness and environmentally benign nature. This material is attractive as a high-capacity anode for battery applications, due to its potential for a 2-electron conversion reaction mechanism, resulting in a high theoretical capacity of approximately 670 mA h g^−1^, nearly doubling the capacity of graphite [39]. The average insertion potential in CuO is approximately ~1.32 V vs. Li^+^/Li, higher than that of graphite but lower than that of Li_4_Ti_5_O_12_ (~1.55 V vs. Li^+^/Li), making it suitable for anodes [39,42]. Numerous publications have addressed the lithium-ion battery (LIB) performance of this material, albeit through methods that may be cost-prohibitive. Consequently, it becomes imperative to revisit this material and unveil its complete potential for LIB applications, utilizing commercially viable techniques [38,39,40,41,43,44,45,46].

In recent years, water-soluble binders have attracted substantial scientific interest as binders for lithium-ion batteries (LIBs), which are imperative for cost-efficient and environmentally sustainable electrode fabrication methodologies [47,48]. Conventionally, *N*-methyl-2-pyrrolidone (NMP) is utilized as a solvent to prepare slurries for cathode or anode materials, a conductive carbon additive, a traditional poly(vinylidene difluoride) (PVDF) binder, and NMP itself. Notably, NMP is classified as a carcinogenic compound with reproductive toxicity, necessitating stringent recycling protocols to mitigate atmospheric contamination [49,50]. Consequently, there is a pronounced shift towards adopting aqueous fabrication processes. Moreover, ongoing research endeavors are extensively investigating the deployment of water-soluble or aqueous binders across both anode and cathode configurations [48,51]. Polymer binders constitute a minor fraction (<5 wt%) of commercial lithium-ion battery (LIB) electrodes, functioning primarily as an adhesive to integrate active materials, conductive agents, and current collectors, thereby preserving both electrical conductivity and mechanical stability within the electrodes [52]. The most commonly employed polymer binders, such as poly(vinylidene difluoride) (PVDF), sodium carboxymethyl cellulose (CMC), and CMC/styrene-butadiene rubber (SBR) blends, have proven effective in graphite anodes due to graphite’s limited volumetric expansion (~10%) upon full lithiation, enabling stable long-term cycling [53,54]. However, in the context of Si-based anodes, PVDF-based binder undergoes significant volume expansion, and the inadequate binding capacity of PVDF results in pronounced silicon particle pulverization, huge expansion, and poor cycling performance. Numerous studies indicate that the cycling stability of Si-based anodes is highly contingent on the choice of polymer binder [55,56,57]. For instance, Si and carbon-coated Si nanoparticle (SiNP) anodes demonstrate markedly improved cycling stability with CMC binders, compared to PVDF. Building on this understanding, our investigation focused on optimizing the performance of a CuO anode by incorporating a CMC binder, enhanced with fluoroethylene carbonate (FEC), as an additive with carbonate-based electrolyte. Comparative analysis was conducted against two binders—CMC and LA133—to evaluate their stabilizing effects on a CuO anode for LIBs.

**Figure 1:**
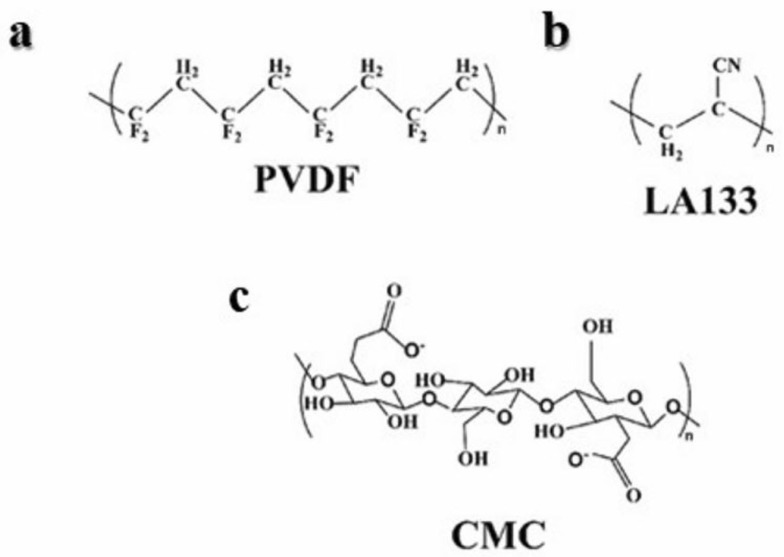
(**a**) PVDF. (**b**) LA133. (**c**) CMC.

The molecular structure of PVDF, CMC, and LA133 are depicted in Figure 1a–c. PVDF is characterized as a linear crystalline polymer with a repeating unit of –CH_2_–CF_2_–. The bonding mechanism of PVDF involves the formation of hydrogen bonds with electrode components, via the fluorine atoms along its extended polymer chains; Carboxymethyl CMC features a backbone comprised of D-glucose residues, interconnected through β-1,4-glycosidic linkages. LA133 is a water-soluble polybasic copolymer, primarily based on polyacrylonitrile. The substantial intermolecular forces in LA133 are attributed to the high polarity of the cyano (–CN) groups within its structure [58]. In contrast, this backbone is modified by carboxymethyl groups (-CH_2_-COOH), attached to some hydroxyl groups of the glucopyranose units, endowing it with high viscosity and pronounced hydrophilicity.

In this study, we utilized high-purity, commercially available CuO compounds, and examined their electrochemical Li-ion insertion performance via a simple solid-state method. Through the optimization of the binder sodium carboxymethyl cellulose (CMC) and electrolyte additives (fluoroethylene carbonate, FEC), we observed a significant enhancement in battery performance. Specifically, the CuO anode demonstrated a remarkable capacity of ~800 mA h g^−1^, an impressive rate capability ~1200 mA g^−1^, and exceptional cycling stability with ~500 cycles (~99% retention). Our findings underscore the potential of CuO as a high-capacity and long-lasting anode material for lithium-ion batteries.

## 2. Result and Discussion

**Figure 2:**
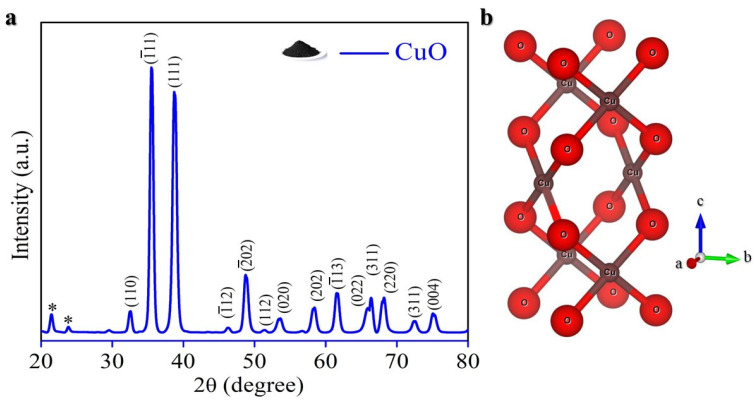
(**a**) XRD pattern; (**b**) Crystal structure.

The commercial CuO nano powder presents as a finely powdered material with a black color Figure 2a (inset). To ascertain its quality and crystalline structure, we conducted X-ray diffraction (XRD) analysis at a scan rate of 2° per minute.

Figure 2a demonstrates that all XRD patterns align closely with the standard CuO compound (JCPDS No. 48-1548, space group C2/c) [59], with minor characteristic peaks indicating traces of impurities (*) from orthorhombic-phase Cu (OH)_2_ (JCPDS No. 13-420) [60]. However, these impurities are negligible, suggesting a predominantly pure CuO formation. The material behaves as a typical p-type semiconductor, featuring a narrow band gap, ranging from 1.2–1.8 eV, which is suitable for LIBs [61]. The crystal structure depicted in Figure 2b exhibits monoclinic CuO, characterized by lattice constants a = 4.6837 Å, b = 3.4226 Å, and c = 5.1288 Å, with angles β = 99.54° and α = γ = 90° [62]. Within this structure, Cu^+^ ions coordinate with four coplanar oxygen atoms arranged at the corners of a rectangular parallelogram, forming chains through edge-sharing [63]. The presence of heavy Cu ions contributes to a high crystal density of 6.31 g cm^−3^, superior to that of graphite, and thus enhancing volumetric energy density [64].

**Figure 3:**
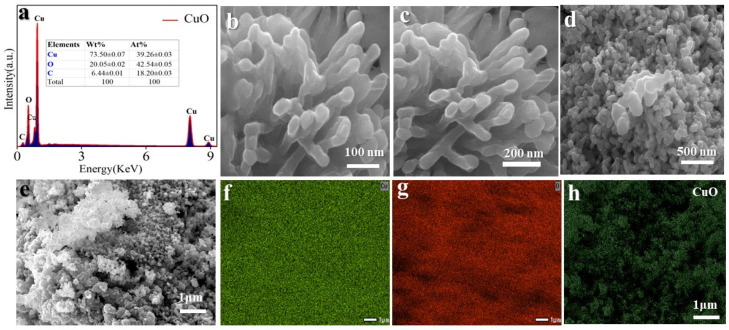
(**a**) EDS patterns; (**b**–**e**) The SEM images from 100 nm to 500 nm and 1 μm, respectively; (**f**–**h**) SEM elemental mapping.

Our investigation extended to the morphology and structural properties of CuO, using scanning electron microscopy (SEM) and Energy dispersive spectroscopy (EDS). Energy dispersive spectroscopy (EDS) analysis at Figure 3a confirms the presence of only Cu and O elements within the sample. Figure 3b–e illustrates the quasi-spherical and mixed morphology of CuO particles, with a size averaging 100–200 nm. Furthermore, elemental mapping results at Figure 3f–h demonstrate homogeneous distribution of Cu and O elements throughout the sample, affirming its purity.

To date, investigations have extensively explored the electrochemical performance of CuO-based anodes in various morphologies, including nanowires [65], nanosheets [66], and nanorods [67], as well as distinctive structures such as mesoporous [68], porous [69], hierarchical [70] core/shell architectures [71], and hollow [72]. Additionally, CuO-based composites have been synthesized by incorporating conductive carbonaceous materials and polymers, such as carbon nanotubes [73], graphene [74], and polypyrrole (PPy) [75], aimed at enhancing electronic conductivity cycles. Furthermore, CuO has been combined with high-capacity anode materials, such as TiO_2_ [76], ZnO [77], SnO_2_ [78], and Fe_2_O_3_ [79] to achieve superior lithium storage capabilities.

Despite considerable efforts dedicated to synthesizing various morphologies and compounds, researchers still fail to fully meet the commercially viable approach and the desirable performance standards of LIBs. Therefore, there persists a need for more extensive research into developing CuO with novel, cost-effective, and commercially viable synthesis techniques, alongside efforts to enhance electrochemical performance. To gain a clearer insight into the current state of research, we outline the testing conditions and performance metrics of reported CuO in Table 1. It is apparent that each of these reports exhibits some limitations, which are shown in Table 1 with our work.

It is widely acknowledged that the selection of binders and electrolyte additives significantly affects the performance of anodes, particularly those made of conversion-alloy, conversion, and alloy-based materials prone to substantial volume expansion [84,85]. Previous studies, as summarized in Table 1, predominantly employed poly(vinylidene fluoride) (PVDF) as the binder, which lacks the ability to effectively accommodate the expansion of electrode volume [86]. Additionally, conventional carbonate electrolytes without additives were commonly used, resulting in the failure to establish a robust solid–electrolyte interphase (SEI) [87]. Therefore, our study focuses on investigating two distinct combinations to evaluate the impact of binders and electrolytes on CuO anode performance: the utilization of CMC (carboxymethyl cellulose) as a non-toxic water-based binder in a regular carbonate electrolyte with fluoroethylene carbonate (FEC) additives [1M LiPF6/EC-DMC + 10% FEC]; and the use of LA133, a non-toxic water-based binder, combined with carbonate electrolyte containing FEC additive [1M LiPF6/EC-DMC + 10% FEC].

**Figure 4:**
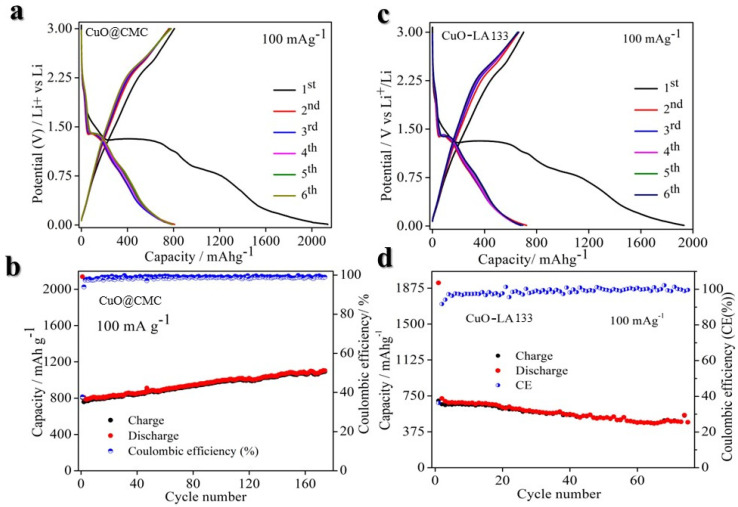
Electrochemical performance of CuO with different binders at a current density of 100 mA g^−1^. (**a**) Galvanostatic charge-discharge (GCD) curve of CuO@CMC. (**b**) Cycling performance of CuO@CMC. (**c**) Galvanostatic charge-discharge curves of CuO@LA133. (**d**) Cycling performance of CuO@LA132.

As depicted in Figure 4a,b, at 100 mA g^−1^ current density, the CuO@CMC electrode initially exhibits a charge capacity of approximately 80 mA h g^−1^ in the first cycle, gradually increasing to ~1100 mA h g^−1^ over 170 cycles. This behavior aligns with prior literature, due to material activation. The average Coulombic efficiency stands at 99%. Notably, the incorporation of the CMC binder with FEC additive demonstrates superior performance, making it the preferred choice for CuO-based anodes. The initial lithiation and delithiation specific capacities are measured at 2138.6 and 807.2 mA h g^−1^, respectively, corresponding to an initial Coulombic efficiency of 37.7%. The significant irreversible capacity loss during the first cycle is largely due to SEI film formation. Notably, the initial lithiation and subsequent specific capacity exceed the theoretical capacity (674 mA h g^−1^) [39], a phenomenon observed in a previous study [39]. In this study, the additional capacity is likely attributed to lithium insertion into a considerable quantity of ketjen black conducting agent or nanopores, interfacial lithium storage, and the formation of SEI.

On the other hand, the CuO@LA133 electrode demonstrates a comparable capacity, with slightly enhanced cycling performance, as illustrated in Figure 4c,d. However, after 75 cycles, the charge capacity and average Coulombic efficiency stabilize around ~375 mA h g^−1^ and 98%, respectively. The LA133 binder’s inadequacy in handling the significant volume changes of CuO anodes leads to particle disintegration, compromising electrical conductivity, and overall performance.

**Figure 5:**
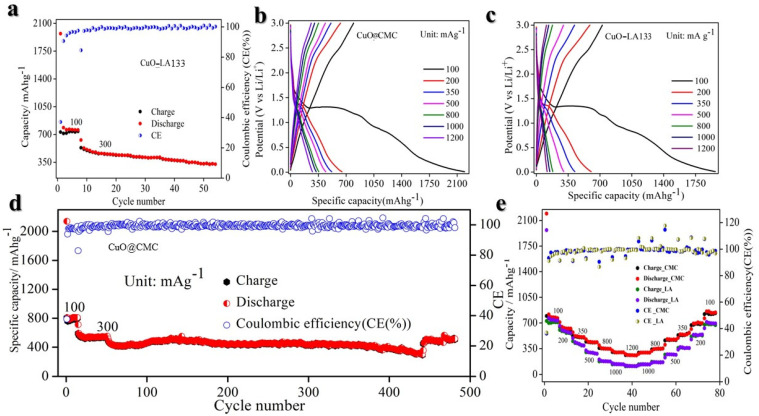
Electrochemical performances of the Li/CuO half-cell in the voltage window of 0.01–3.0 V vs. Li^+^/Li. (**a**) Cycling performance of CuO@LA133 at 300 mA g^−1^. (**b**) Rate curve of CuO@CMC from 100–1200 mA g^−1^ current density. (**c**) Rate curve of CuO@LA132 from 100–1200 mA g^−1^ current density. (**d**) Cycling performance of CuO@CMC at 300 mA g^−1^. (**e**) Comparison of rate performance of CuO@CMC and CuO@LA133 binders.

As depicted in Figure 5a, the CuO@LA133 electrode exhibits an initial charge capacity of approximately 700 mA h g^−1^ at low current density of 100 mA g^−1^, as shown in Figure 4d. This capacity remains stable at 700 mA h g^−1^ for the first 7 cycles. However, when subjected to a higher current density of 300 mA g^−1^, the capacity rapidly declines to about 300 mA h g^−1^ over 54 cycles. The average Coulombic efficiency observed is 98.31%. This indicates that the LA133 binder is inadequate in accommodating the significant volumetric changes of the CuO anodes, failing to maintain particle cohesion, which is crucial for preserving electrical conductivity and electrochemical stability.

In contrast, as shown in Figure 5d, the CuO@CMC electrode demonstrates an initial charge capacity of around 800 mA h g^−1^, maintaining this capacity for the first 7 cycles. Upon increasing the current density to 300 mA g^−1^, the electrode maintains a stable capacity of approximately 450 mA h g^−1^ over ~500 cycles, with an average Coulombic efficiency of 99.10%. This superior performance highlights the effectiveness of the CMC binder in managing the volumetric expansion of CuO anodes, ensuring particle integrity, sustained electrical conductivity, and better electrochemical performance.

**Figure 6:**
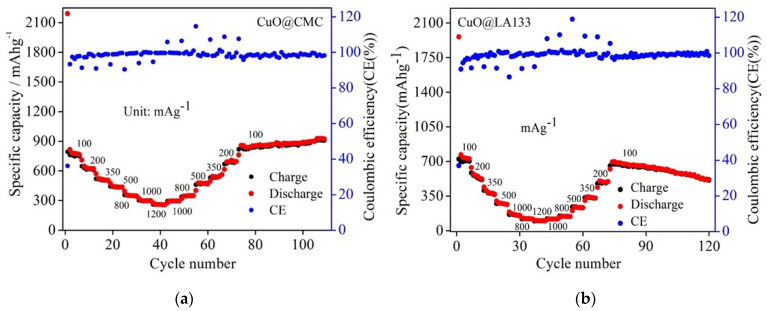
(**a**) CuO with CMC based electrode. (**b**) CuO with LA133 based electrode.

In addition to exhibiting high capacity and stable performance, the CuO@CMC anode demonstrates superior rate performance and long-term cycling stability. As illustrated in Figure 5b, the charge capacities of CuO@CMC-based electrodes are 761, 618, 515, 435, 345, 297, and 258 mA h g^−1^ at current densities of 100, 200, 350, 500, 800, 1000, and 1200 mA g^−1^, respectively. Notably, even at 1200 mA g^−1^ current density, the charge capacity remains at 258 mA h g^−1^, which is comparable to that of graphite. Upon reverting the current density to 1000, 800, 500, 350, 200, and 100 mA g^−1^, the charge capacities recover to 294, 350, 477, 544, 693, and 853 mA h g^−1^, respectively, achieving a recovery ratio of approximately 100%, indicative of excellent rate performance as depicted in Figure 5e, and in separate Figure 6a. Conversely, as shown in Figure 5c, the charge capacities of CuO@LA133-based electrodes are 698, 562, 420, 295, 178, 137, and 111 mA h g^−1^ at current densities of 100, 200, 350, 500, 800, 1000, and 1200 mA g^−1^, respectively. When the current density is reduced back to 1000, 800, 500, 350, 200, and 100 mA g^−1^, the charge capacities recover to 135, 164, 266, 355, 530, and 681 mA h g^−1^, respectively, corresponding to a recovery ratio of approximately 98%. However, this exhibits inferior rate performance, as shown in Figure 5e, and in separate Figure 6b.

**Figure 7:**
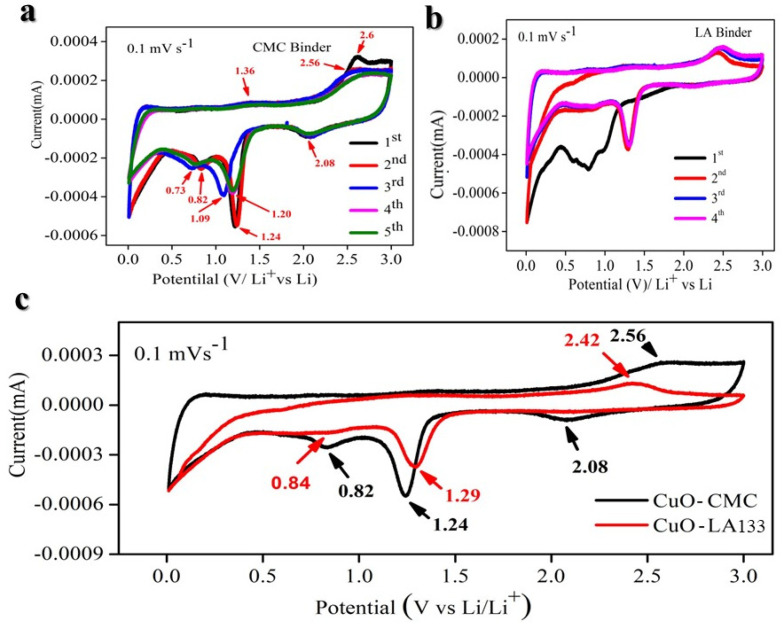
Cyclic voltammetry (CV). (**a**) CuO@CMC based electrode. (**b**) CuO@LA133 based electrode. (**c**) Comparison of CV of CuO@CMC and CuO@LA133 based electrodes.

To obtain a more detailed understanding of the Li-insertion mechanism in CuO using both CMC and LA133 binders, we performed a cyclic voltammetry (CV) analysis. Figure 7a illustrates the cyclic voltammetry (CV) profiles obtained at a scan rate of 0.1 mV s^−1^ within a potential window of 0.01–3.0 V. During the initial cathodic sweep, three distinct reduction peaks are observed at 2.08, 1.24, and 0.82 V. These peaks correspond to the stepwise reduction of CuO: the first peak represents the reduction of CuO to an intermediate phase (${Cu}_{1-x}^{II}{Cu}_{x}^{I}O_{1-x/2}$ (0 ≤ x ≤ 0.4), the second peak indicates further reduction to Cu_2_O, and the third peak signifies the conversion to metallic Cu and Li_2_O. In the subsequent anodic sweep, oxidation peaks appear at 1.36, 2.56, and 2.6 V, which are associated with the oxidation of Cu back to Cu_2_O and the reformation of CuO [44]. The reaction mechanisms involved are described by their respective electrochemical equations [44]:

Discharging

(1) $$CuO+x{\mathrm{Li}}^{+}+xe^{-}\to \;{Cu}_{1-x}^{II}{Cu}_{x}^{I}O_{1-x/2}+x/2{Li}_{2}O\;(0\;\leq \;x\;\leq \;0.4)$$

(2) $${Cu}_{1-x}^{II}{Cu}_{x}^{I}O_{1-x/2\;}+(1-x)\;{\mathrm{Li}}^{+}+(1-x)e^{-}\to \;{\mathrm{Cu}}_{2}O+(1-x)/2{\mathrm{Li}}_{2}O\;(0\;\leq \;x\;\leq \;0.4)$$

1/2Cu_2_O + Li^+^ + e^−^ → Cu + 1/2Li_2_O (3)

Charging

Cu + 1/2Li_2_O → 1/2Cu_2_O + Li^+^ + e^−^ (4)

1/2Cu_2_O + 1/2Li_2_O → CuO^+^ + Li^+^ + e^−^ (5)

The difference in peak areas between the first cycle and subsequent cycles can be attributed to the formation of the solid-electrolyte interface (SEI) film and electrolyte decomposition [88]. A slight positive shift in potential observed during the second and third cycles indicates structural rearrangements occurring during the initial lithium-ion insertion [39,43]. From the second cycle onwards, the CV curves demonstrate consistent reproducibility, highlighting the superior electrochemical reversibility of the CuO@CMC (Figure 7a) electrode relative to the CuO@LA133 (Figure 7b) electrode, as can be observed in the comparative CV curve in Figure 7c. Figure 4a showcases the charge/discharge voltage profiles for the first six cycles at a current density of 100 mA g^−1^, revealing three distinct voltage plateaus (2.0–1.28, 1.29–1.26, and 1.27–0.03 V) during the initial discharge, which corroborate the multi-step conversion process of CuO to Cu in the presence of Li, as reflected in the CV results.

**Figure 8:**
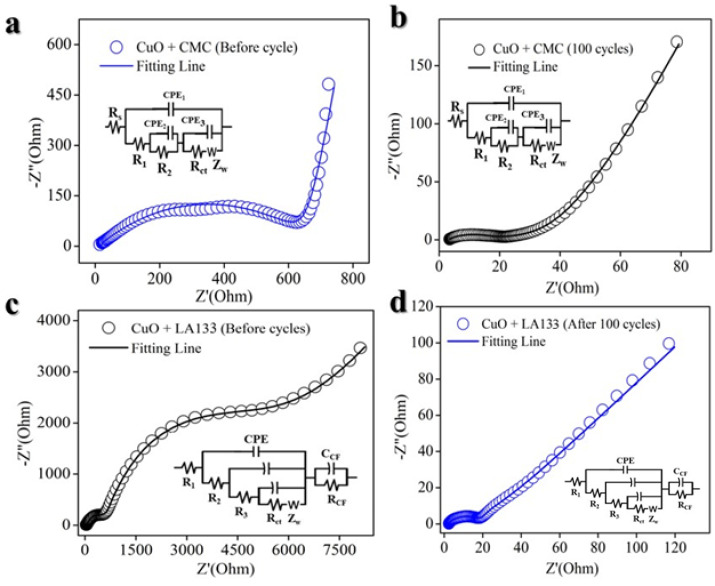
Electrochemical impedance spectroscopy. (**a**) CuO@CMC before cycling. (**b**) CuO@CMC after 100 cycles. (**c**) CuO@LA133 before cycle. (**d**) CuO@LA133 after 100 cycles.

From an electrochemical perspective, Electrochemical Impedance Spectroscopy (EIS) was utilized to elucidate the kinetic processes governing the electrode reactions. Figure 8a and 8c present the Nyquist plots for the CuO@CMC and CuO@LA133 electrodes, respectively, prior to cycling at a current density of 100 mA g^−1^. Both spectra are characterized by the presence of two semicircles and a sloping line. Notably, the CuO@LA133 electrode exhibits a significantly larger semicircle prior to cycling. As depicted in Figure 8c, charge transfer resistance (Rct) is 3.031 × 10^3^ Ω, indicating slower kinetics in the CuO@LA133 electrode. The semicircles observed at high and high-to-medium frequencies correspond to the resistance of the solid electrolyte interphase (SEI) film and the Rct at the electrode/electrolyte interface, respectively. But, as shown in Figure 8a, CuO@CMC electrode Rct value is 2.30 × 10^2^ Ω, which is lower than that of LA133 based electrode. The sloping line at low frequencies is indicative of Li⁺ diffusion within the solid state. Importantly, the CuO@CMC electrode (Figure 8b) after 100 cycles demonstrates the smallest semicircle diameter before cycling, signifying the lowest Rct value 21 Ω relative to the CuO@LA133 electrode (Figure 8d) Rct values is 77 Ω. This observation suggests that the CuO@CMC electrode exhibits the lowest polarization and the most rapid reaction kinetics.

**Figure 9:**
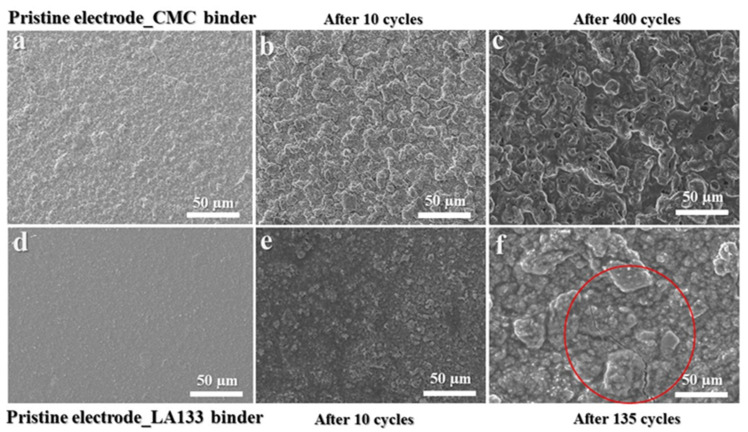
Top-view SEM images Pre- and Post-Cycle Comparisons. (**a**) Prestin CuO@CMC electrode. (**b**) CuO@CMC electrode after 10 cycles. (**c**) CuO@CMC electrode after 400 cycles. (**d**) Prestin CuO@LA133 electrode. (**e**) CuO@LA133 electrode after 10 cycles. (**f**) CuO@LA133 electrode after 135 cycles.

Furthermore, SEM analysis was employed to assess the morphological analysis of CuO@CMC and CuO@LA132 electrodes both prior to and following cycling. As depicted in Figure 9a–c, the CuO@CMC electrode exhibits no cracks before cycling, after 40 cycles, and even after 400 cycles. Notably, the SEM image in Figure 9d reveals a smooth, gel-like film on the surface after 400 cycles, indicating increased stability and density.

This improved morphology enhances the electrochemical environment, leading to extended cell lifespan, improved storage performance, and overall superior electrochemical performance. Conversely, Figure 9d–f illustrate the CuO@LA133 electrode before cycling and after 10 cycles, with no visible cracks. However, by 135 cycles, a significant crack is observed, as highlighted by the red circle in Figure 9f, demonstrating that the LA133 binder is not suitable for CuO anode material in LIBs.

The enhanced performance of CuO@CMC electrodes in lithium-ion batteries (LIBs) can be attributed to several factors. The hydrophilic nature of CMC enhances the electrode’s wettability with the electrolyte, resulting in improved ionic conductivity, and more efficient ion transport within the electrode. This reduces internal resistance and boosts the battery’s overall performance. CMC’s superior mechanical flexibility, compared with PVDF, allows it to accommodate the volume changes and stress associated with CuO’s lithiation and delithiation processes [89,90,91]. This flexibility minimizes electrode cracking and maintains structural integrity, leading to longer cycle life and stable performance. Moreover, CMC ensures a homogeneous distribution of CuO particles within the electrode matrix, preventing agglomeration and promoting consistent and efficient electrochemical reactions throughout the electrode, thereby enhancing capacity and efficiency [48,91]. CMC also forms a conductive network that improves electron transport within the electrode, facilitating efficient electron transfer during charge and discharge processes. This contributes to higher rate capabilities and overall better electrochemical performance [92,93]. The strong adhesion provided by CMC between CuO particles and the current collector, as well as among the active materials, maintains electrode integrity during cycling, reducing the likelihood of active material detachment and capacity loss [47,94]. CMC’s compatibility with aqueous processing minimizes side reactions often associated with organic solvents used in PVDF-based electrodes, leading to a more stable electrochemical environment and extending the battery’s lifespan. Additionally, the use of water as a solvent in CMC-based electrodes eliminates the need for hazardous organic solvents like NMP, making the manufacturing process safer, more environmentally friendly, and cost-effective. In brief, the scientific reasons for the improved performance of CuO@CMC electrodes in LIBs include enhanced ionic and electron conductivity, superior mechanical flexibility, improved dispersion of active materials, stronger adhesion, and minimized side reactions. These factors collectively contribute to higher capacity, better cycle stability, and overall improved electrochemical performance. Furthermore, the environmental and cost benefits associated with aqueous processing make CMC a highly attractive binder for advanced LIB applications. It is well known that the incorporation of fluoroethylene carbonate (FEC) as an additive in lithium-ion battery (LIB) anodes brings significant advantages, particularly in enhancing performance and extending battery lifespan. A critical benefit is the promotion of a stable and uniform solid electrolyte interphase (SEI) layer, which is especially important for graphite- and silicon-based anodes. This robust SEI layer mitigates continuous electrolyte decomposition, thereby improving battery efficiency. Moreover, the enhanced stability of the SEI with FEC contributes to superior cycling stability by minimizing active lithium loss, a crucial factor for high-capacity anodes like silicon that experience substantial volume expansion. FEC also improves the first-cycle Coulombic efficiency by reducing lithium consumption during initial SEI formation, leading to better overall battery performance. Additionally, it reduces capacity fade over extended cycles by stabilizing the SEI and limiting electrolyte degradation, thus helping the battery maintain its capacity across numerous charge–discharge cycles. Another key advantage is the suppression of lithium dendrite formation, a critical concern in high-energy-density anodes, thereby lowering the risk of short circuits, and enhancing battery safety. Finally, FEC is compatible with high-voltage electrolyte systems, making it well-suited for advanced LIB chemistries that operate at elevated voltages, which in turn increases energy density.

## 3. Materials and Methods

### 3.1. Synthesis

Commercially available CuO nano powder (Sigma-Aldrich, 99.99% purity, Rockville, MD, USA) was utilized as the anode material for this experimental investigation, without any additional modifications.

### 3.2. Characterization

Structural examination of the CuO nano powder was conducted using an X-ray diffractometer (Rigaku SmartLab, The Woodlands, TX, USA), equipped with Cu Kα radiation (λ = 1.5408 Å), over a range from 20–80° at a scan rate of 2° per minute. Additionally, scanning electron microscopy (SEM) and energy-dispersive X-ray spectroscopy (EDS) were employed, equipped with a backscattered secondary electron detector (SEI) operating at an accelerating voltage of 20 kV and Dry SD_30_ detector for morphological examination and chemical composition identification, respectively, utilizing a JEOL JSM 6480LV instrument (JEOL, Akishima, Tokyo).

### 3.3. Cell Fabrication & Electrochemical Measurement

To conduct the electrochemical testing, the anode was prepared by mixing 50% (*w*/*w*) of the active material, CuO, with 25% (*w*/*w*) Ketjen black as a conducting agent, and 25% (*w*/*w*) binder [either LA133 or CMC]. Both slurries were prepared using water as a solvent. The resulting slurries were spread onto a copper foil (9 μm thickness, MTI Corporation, Richmond, CA, USA), using a doctor blade (MTI Corporation, Richmond, CA, USA). The coated foil was then dried overnight at 60 °C for 16 h. Anodes with a diameter of 1 cm were punched (MTI Corporation, Richmond, CA, USA) from the dried coated foil, and further dried at 60 °C under vacuum for 12 h before being transferred into the glovebox (MBRAUN Glovebox Workstations, Stratham, NH, USA). The average active mass loading of electrodes is in the range of 1.2–1.7 mg. For assembling CR2032 coin-type half-cells, lithium chips (MSE Supplies LLC, Tucson, AZ, USA) were used as the counter electrode, and polypropylene ethylene was used as the separator. Regarding the electrolyte, a commercial solution of 1 M LiPF_6_ in ethylene carbonate (EC) and diethyl carbonate (DEC) [1:1] with 10% fluoroethylene carbonate (FEC) additives was prepared. Subsequently, the electrochemical performance of CuO for both LA133 and CMC-based electrodes was evaluated. We employed the Landt battery tester (LANDT Instruments, Vestal, NY, USA) to conduct electrochemical measurements, setting the voltage range at 0.01–3.0 V. Cyclic voltammetry (CV) was performed within the same range, while electrochemical impedance spectroscopy (EIS) was measured using the Arbin tester (Arbin Instruments, College Station, TX, USA) across a frequency range from 0.01 Hz to 100 kHz.

## 4. Conclusions

In this study, we introduce an efficient and cost-effective method for producing CuO electrode material. CuO@CMC based electrodes demonstrate remarkable electrochemical performance as compared to CuO@LA133 based electrodes, revealing a high specific capacity of approximately ~1100 mA h g^−1^ at a current density of 100 mA g^−1^ after 170 cycles, impressive rate performance of around 325 mA h g^−1^ at high current density 1200 mA g^−1^, and excellent long-term stability of about 450 mA h g^−1^ at 300 mA g^−1^ for over ~500 cycles. By employing CuO nanoparticles in combination with a suitable amount of high surface area conducting ketjen black carbon, and an appropriate amount of CMC binder, along with the addition of 10% FEC additive to a regular carbonate-based electrolyte, we effectively address the volume expansion issue encountered in CuO anodes for lithium-ion batteries (LIBs). Additionally, this strategy enhances both electron and ion conductivity. Our findings offer a novel and facile approach for manufacturing transition metal oxide (TMO) anode materials with enhanced energy density, thereby facilitating their widespread adoption in advanced energy storage technologies.

## Figures and Tables

**Table 1:** Comparative analysis of this work with previously reported CuO-based anode materials.

Ref.	Samples	Rate	Cycles	Capacity	Notes
[80]	Hollow CuO nanoparticles	100	100	630	Time and energy consuming and expensive synthesis route
[46]	CuO/Cu_2_O/C composites	200	600	260	Energy consuming method
[81]	Cu_x_O/C anode	100	100	335	Time and energy consuming
[82]	CuO/tube-like carbon	100	100	650	Complicated and expensive synthesis approach
[83]	CuO@Cu microspheres	100	100	876	Sensitive route and time consuming
[65]	CuO nanowire arrays	300	100	550	Complicated synthesis approach
[74]	CuO nanosheets	100	100	600	Poor performance and expensive method
[39]	CuO@C	100 500	100 700	1024	Toxic and energy consuming process
[44]	Peony shaped CuO nanosheets	100 1000	80 100	780 441	Time consuming
This work	Commercial CuO nano powder	100 300	170 500	800 450	Safe, simple, cost-effective, scalable, and financially sustainable strategy for battery production.

## Data Availability

The data presented in this study are available on request from the corresponding author.
